# Biopolymer-Based 3D Printing for Dental–Pulp Complex Tissue Regeneration: Innovations and Challenges

**DOI:** 10.3390/molecules31132262

**Published:** 2026-06-26

**Authors:** Loredana Corina Toderici, Claudia Nicoleta Feurdean, Alexandrina Muntean, Dana Feștilă, Sanda Mihaela Popescu, Anca Ionel, Radu Chifor, Anida Maria Băbțan, Willi Andrei Uriciuc, Aranka Ilea

**Affiliations:** 1 Department of Oral Rehabilitation, Faculty of Dentistry, “Iuliu Hațieganu” University of Medicine and Pharmacy, 400012 Cluj-Napoca, Romania; toderici_lore_corina@elearn.umfcluj.ro (L.C.T.); ionel.anca@umfcluj.ro (A.I.); chifor.radu@umfcluj.ro (R.C.); babtan.anida@umfcluj.ro (A.M.B.); 2 Department of Paediatric Dentistry, Faculty of Dentistry, “Iuliu Hațieganu” University of Medicine and Pharmacy, 400012 Cluj-Napoca, Romania; alexandrina.muntean@umfcluj.ro; 3 Department of Orthodontics, Faculty of Dentistry, “Iuliu Hațieganu” University of Medicine and Pharmacy, 400012 Cluj-Napoca, Romania; dana.festila@umfcluj.ro; 4 Department of Oral Rehabilitation, Faculty of Dentistry, University of Medicine and Pharmacy of Craiova, 200349 Craiova, Romania; sanda.popescu@umfcv.ro; 5 Faculty of Nursing and Health Sciences, “Iuliu Hațieganu” University of Medicine and Pharmacy, 400012 Cluj-Napoca, Romania; willi.uriciuc@umfcluj.ro

**Keywords:** biopolymers, 3D printing, tissue engineering, regenerative dentistry, pulp regeneration, scaffolds, bioink

## Abstract

The regeneration of the dentin-pulp complex remains a significant challenge in regenerative endodontics. While conventional therapeutic approaches are effective in eliminating infection and preserving dental structure, they fail to restore the biological functionality of the pulp tissue. In recent years, three-dimensional (3D) printing and biopolymer-based bioprinting have opened unprecedented opportunities in dental tissue engineering, enabling the fabrication of biomimetic scaffolds with precisely controlled structural and bioactive properties. This review synthesizes current advances in bioprinting technologies, the diversity of biomaterials and bioinks employed, and the various stem cell sources utilized in pulp regeneration. It further examines how the three-dimensional microenvironment modulates cell viability, odontogenic differentiation, and the promotion of angiogenesis and neurogenesis, emphasizing the role of scaffold composition, mechanical properties, and internal architecture in influencing regenerative outcomes. Additionally, persistent challenges are discussed, including the optimization of bioink formulations, the achievement of functional vascular integration, and long-term validation of regenerated tissues, underscoring the need for multidisciplinary strategies to facilitate clinical translation. By integrating recent evidence, this review establishes a conceptual framework for the development of personalized and predictable approaches to dentin-pulp complex reconstruction.

## 1. Introduction

Although substantial advances have been achieved in endodontic therapy, the regeneration of a fully functional dentin–pulp complex remains one of the most challenging and unresolved goals in dental tissue engineering. Conventional endodontic treatments are highly effective in eliminating infection and preserving dental structure; however, they fail to restore the biological functionality of the dental pulp, including sensory perception, immune defense, vascularization, and physiological dentin formation. Consequently, regenerative strategies aimed at reconstructing a functional dentin–pulp unit have become a central focus of contemporary dental research [1,2,3]. In the context of regenerative endodontics, functional dentin–pulp complex regeneration should not be defined solely by mineralized tissue deposition or odontogenic marker expression, but rather by the re-establishment of a vascularized, innervated, and immunocompetent pulp tissue capable of maintaining sensory function, metabolic homeostasis, immune defense, and physiological dentin remodeling over time. Importantly, many currently available studies primarily demonstrate early biological responses and structural tissue formation, whereas long-term functional integration and physiological performance remain insufficiently validated [3,4,5]. Research in regenerative medicine has increasingly focused on the development of advanced biomaterials and fabrication strategies capable of restoring tissue architecture and functional integration, moving beyond the mere replacement of lost structures. In this context, biomimetic three-dimensional (3D) scaffolds have attracted growing attention due to their ability to provide not only structural support but also biochemical and biophysical cues that regulate cell proliferation, differentiation, and spatial organization [4,5,6]. Ideally, such constructs should combine high biocompatibility with controlled biodegradation kinetics, allowing the gradual replacement of the scaffold with newly formed tissue while maintaining mechanical stability throughout the regenerative process [7,8].

**Figure 1:**
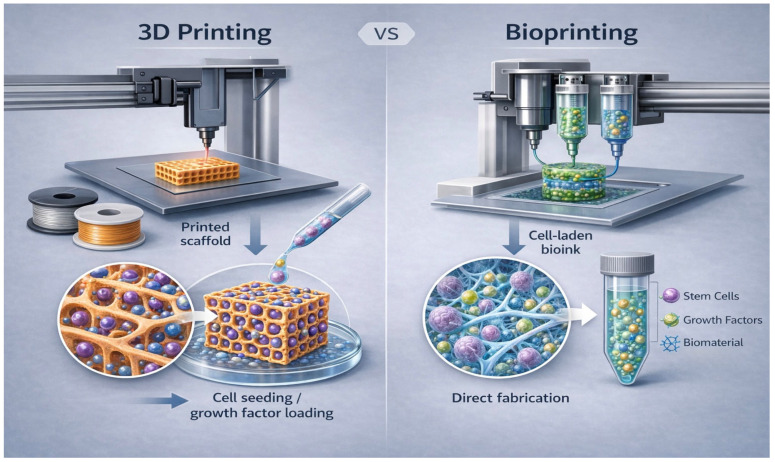
Schematic comparison between 3D printing and bioprinting approaches.

The fabrication of scaffolds for dental tissue engineering has evolved considerably over recent decades. Early approaches relied on conventional processing techniques such as electrospinning, freeze-casting, gas foaming, and solvent casting [9,10,11,12]. Although these methods have contributed to the development of scaffolds capable of supporting tissue regeneration, they generally offer limited control over internal scaffold architecture, particularly regarding spatial organization and pore uniformity. Moreover, these processes may pose challenges in removing residual compounds and do not consistently enable accurate reproduction of the complex geometries required to mimic native tissues [13]. To overcome these limitations, additive manufacturing technologies, particularly 3D printing and bioprinting [14], have emerged as transformative tools in dental tissue engineering (Figure 1).

**Figure 2:**
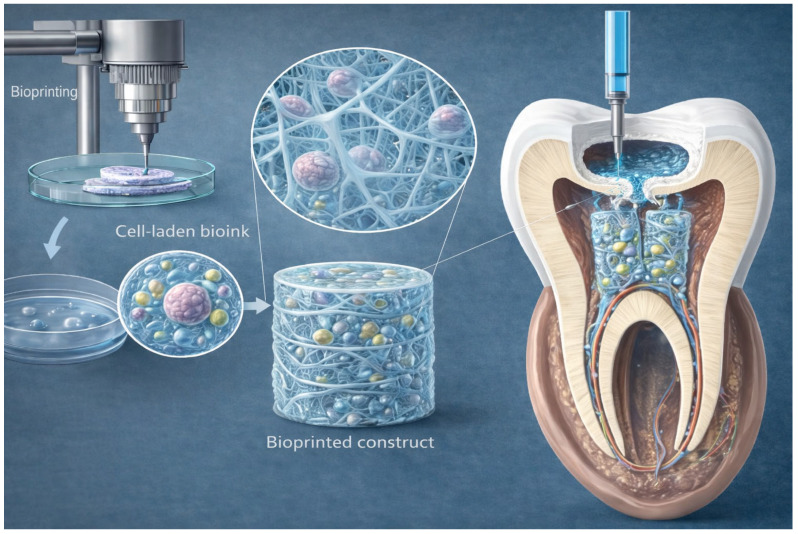
Schematic representation of bioprinted scaffold application for dentin–pulp complex regeneration.

These techniques enable the layer-by-layer fabrication of complex, patient-specific constructs with tunable porosity, geometry, and functional properties. Although bioprinting is an extension of conventional 3D printing, the two approaches differ fundamentally in their design philosophies. Traditional 3D printing primarily focuses on the fabrication of mechanically stable scaffolds that are subsequently seeded or functionalized with biological components, whereas bioprinting integrates living cells, growth factors, and bioactive materials directly during the fabrication process [15,16,17], enabling the creation of biologically active and spatially organized constructs (Figure 2).

Despite significant progress in scaffold design, biomaterial development, and stem cell-based therapies, achieving predictable regeneration of a fully functional dentin–pulp complex remains a significant challenge. In particular, the coordinated regulation of multiple biological processes, including vascularization, innervation, and organized dentinogenesis within engineered three-dimensional environments, remains difficult to achieve. Furthermore, discrepancies between promising in vitro findings and the variable outcomes observed in vivo underscore the need for a comprehensive synthesis of current fabrication strategies and their biological performance. Emerging evidence suggests that regenerative success is determined less by the printing modality itself and more by the engineered three-dimensional microenvironment that governs stem cell fate, tissue integration, and long-term functional stability. Understanding how scaffold composition, mechanical properties, architectural organization, and cellular distribution collectively influence regenerative outcomes is therefore essential for advancing translational endodontic therapies. Accordingly, this review aims to critically evaluate recent advances in the application of 3D printing and bioprinting for dentin–pulp complex regeneration, with particular emphasis on scaffold fabrication techniques, biomaterial selection, stem cell sources, and the biological outcomes reported in both in vitro and in vivo studies. By synthesizing current evidence, identifying key challenges, and highlighting emerging opportunities, this review seeks to establish a robust conceptual framework for future progress in regenerative endodontics.

## 2. Fabrication Techniques for 3D Scaffolds with Regenerative Potential

The regenerative performance of 3D scaffolds is profoundly influenced by the fabrication strategy employed, as processing parameters directly determine internal architecture, cellular distribution, mechanical properties, and post-implantation biological behavior. In this context, the development of additive manufacturing technologies has enabled unprecedented control over construct geometry, facilitating the design of biomimetic structures capable of supporting cellular organization, vascular infiltration, and guided tissue regeneration [18,19]. In both 3D printing and bioprinting, the creation of a digital model is an essential prerequisite prior to fabrication. These models can be generated using computer-aided design (CAD) software or derived from medical imaging techniques such as computed tomography (CT), magnetic resonance imaging (MRI), or radiography, thereby enabling the precise reproduction of the desired three-dimensional morphology [20,21].

**Figure 3:**
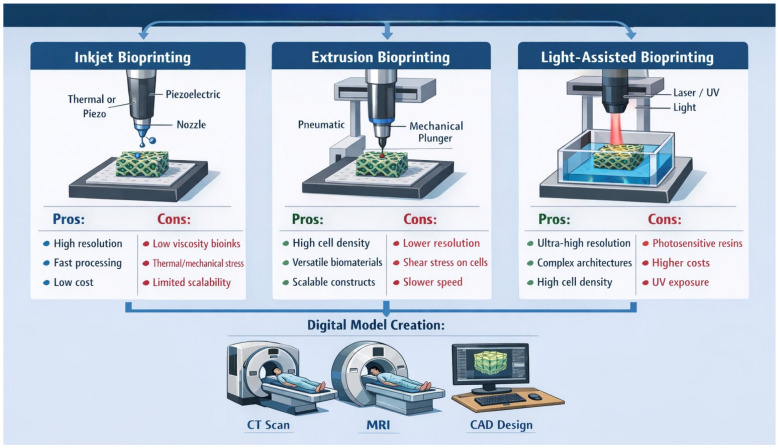
Schematic comparison of the main advantages and limitations of 3D scaffold fabrication techniques.

Current 3D printing technologies can be broadly classified into three main categories based on the material deposition mechanism: inkjet printing (IJP), extrusion-based printing (EBP), and light-assisted printing (LAP) (Figure 3). These systems have subsequently been adapted for bioprinting applications to enable the processing of biomaterials and living cells while maintaining the core operational principles of conventional printing platforms [19].

### 2.1. Inkjet-Based Bioprinting

Inkjet-based bioprinting (IJP) relies on the controlled generation of biomaterial droplets through thermal or piezoelectric actuation. In thermal systems, rapid heating induces bubble formation, facilitating the expulsion of material through the nozzle, whereas piezoelectric systems employ pressure waves to generate discrete droplets [22,23]. This technique offers high processing speed and superior spatial resolution compared to extrusion-based methods, making it particularly suitable for generating fine cellular patterns and replicating tissue microarchitecture. Additionally, it is characterized by high operational flexibility and relatively low cost. However, the requirement for low viscosity bioinks limits both achievable cell density and the mechanical stability of the resulting constructs [24]. Furthermore, exposure to thermal and mechanical stresses may negatively affect cell viability, droplet uniformity, and cell encapsulation efficiency [25]. Compared to LAP, IJP-based bioprinting exhibits lower scalability and limited capacity for generating volumetric structures, which represents a significant constraint for endodontic applications requiring complete root canal filling. Consequently, this method is best suited for the fabrication of functional microstructures or localized cell delivery systems, although its translation to large-scale clinical applications requires further optimization of bioink rheology and post-printing stability [17,26].

### 2.2. Extrusion-Based Bioprinting

Extrusion-based bioprinting (EBP) is one of the most widely used technologies in regenerative dental medicine due to its material versatility, scalability, and accessibility [27]. This approach utilizes either pneumatic systems driven by compressed air or mechanical systems based on pistons or screws, enabling continuous material deposition through a nozzle [28]. Printing platforms are typically equipped with thermally controlled dispensing systems and movable stages that allow positioning along the x, y, and z axes, facilitating the fabrication of complex three-dimensional constructs. Technologies such as fused deposition modeling (FDM) and direct ink writing (DIW) are based on this controlled extrusion principle [29].

A major advantage of EBP is its ability to process high-viscosity biomaterials and accommodate elevated cell densities, enabling the fabrication of large and scalable constructs compared to other methods limited by rheological constraints [23]. However, the resolution of extrusion-based constructs is generally lower than that of other techniques, and the extrusion of highly viscous materials may subject cells to shear stress, adversely affecting viability [30]. Consequently, bioink selection is critical for maintaining structural fidelity after deposition and ensuring overall construct integrity. Although low viscosity bioinks are more challenging to process using this method, support bath printing strategies have emerged as a promising solution to overcome these limitations [31,32]. This approach is particularly attractive for dental applications due to its scalability and flexibility in bioink design. Nevertheless, optimization of shear-thinning behavior remains essential to ensure both cell survival and geometric fidelity, especially in the context of complex dentin-pulp structures.

### 2.3. Light-Assisted Bioprinting

Light-assisted bioprinting (LAP) is based on the photopolymerization of materials under laser or light exposure, enabling the fabrication of high-resolution three-dimensional constructs. This category includes several techniques, such as Laser-Induced Forward Transfer (LIFT), stereolithography (SLA), and Digital Light Processing (DLP). In LIFT, a laser pulse transfers biomaterial onto a target substrate; SLA uses a laser or ultraviolet (UV) light to selectively solidify a photosensitive polymer; and DLP builds upon SLA by projecting digital light patterns to polymerize entire layers simultaneously [6,19]. As these methods do not rely on nozzles, issues related to clogging are eliminated, allowing the processing of a broader range of bioink viscosities. Light-assisted techniques offer exceptionally high resolution, precise spatial control over cell placement, and the ability to fabricate constructs with high cell densities (up to 1 × 10^8^ cells/mL), resulting in excellent structural fidelity [26]. These approaches have shown considerable promise in dental applications, including dental pulp and tooth germ regeneration, by supporting epithelial–mesenchymal interactions and promoting pulp revascularization [19].

Despite these advantages, several limitations remain. Material selection is restricted to photosensitive polymers, often requiring chemical modification to ensure compatibility with photopolymerization processes. Additionally, photopolymers are distributed throughout the entire resin reservoir, including non-target regions, potentially leading to material waste and increased costs. The presence of photoinitiators and free radicals may also adversely affect cell viability, while exposure to UV light can induce cellular stress [6,26]. Overall, LAP represents a powerful approach for the fabrication of high-precision, high-cell-density scaffolds, particularly in the context of dental pulp regeneration. Continued optimization of bioink composition and the development of biocompatible photopolymerization systems are essential for successful clinical translation.

Although all major bioprinting modalities have demonstrated regenerative potential, their translational relevance differs substantially according to the balance between structural fidelity, scalability, cell viability, and biomaterial compatibility. Inkjet-based systems provide superior spatial resolution and precise cell patterning but remain limited by low-viscosity bioinks and reduced mechanical stability, restricting their applicability in large volumetric pulp constructs [17,24]. In contrast, extrusion-based bioprinting currently appears more clinically adaptable due to its scalability, ability to process high-viscosity biomaterials, and compatibility with cell-laden hydrogels and hybrid scaffolds [23]. However, its lower resolution and shear stress-associated effects on cells remain important limitations [30]. Light-assisted approaches provide exceptional architectural precision and high cell density but are constrained by the need for photopolymerizable materials and concerns regarding photoinitiator cytotoxicity and long-term safety [6,26]. Consequently, no single printing modality currently fulfills all biological and translational requirements for dentin–pulp complex regeneration, suggesting that future progress will likely rely on hybrid fabrication strategies combining architectural precision with biologically instructive microenvironments. Current evidence further suggests that scaffold bioactivity and microenvironmental regulation may outweigh the isolated contribution of fabrication modality itself in determining regenerative outcomes.

## 3. Materials Used in the Fabrication of 3D Scaffolds with Regenerative Potential for the Dentin-Pulp Complex

**Figure 4:**
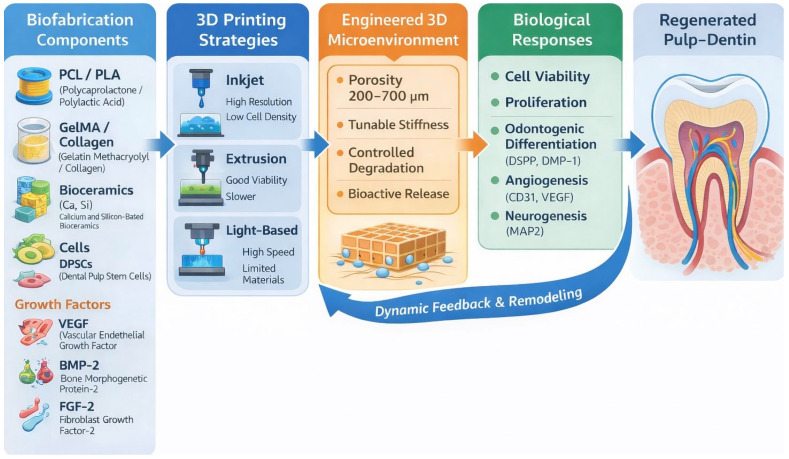
Schematic representation of the bioprinting process and its main features.

Within 3D printing technologies applied to dental tissue regeneration, a broad range of biomaterials can be utilized, including synthetic polymers, ceramic materials, hydrogels, and various composite systems [19]. Given that these scaffolds are frequently seeded with stem cells or loaded with bioactive molecules, they must exhibit adequate biocompatibility, controlled biodegradability, and structural properties that support three-dimensional cellular organization, vascularization, and nutrient diffusion. Porosity represents a critical parameter for facilitating cell adhesion and proliferation, while the incorporation of growth factors or other bioactive agents can accelerate differentiation and tissue maturation. Furthermore, material selection enables the tuning of mechanical properties and degradation kinetics, allowing scaffolds to provide temporary structural support while being gradually resorbed and replaced by host tissue. In this context, both biodegradable synthetic polymers and extracellular matrix (ECM)-derived materials, as well as composite systems [33,34], have demonstrated significant potential for the development of functional constructs for pulp–dentin tissue engineering (Figure 4).

### 3.1. Biodegradable Synthetic Polymers

Polycaprolactone (PCL) is one of the most widely used synthetic polymers in three-dimensional bioprinting, including dental regenerative applications, due to its favorable mechanical properties, biocompatibility, and relatively low melting temperature (approximately 60 °C), which enables controlled extrusion through fine nozzles. Additionally, its long-term structural stability makes it suitable as an implantable material capable of maintaining scaffold integrity throughout tissue remodeling [35]. However, the use of PCL as a standalone material is limited by its hydrophobic nature and slow degradation rate, both of which are associated with reduced cell adhesion and bioactivity. To address these limitations, numerous studies have explored hybrid systems combining PCL with bioactive materials such as calcium-based bioceramics, bioactive glasses (BG), ECM-derived components, and hydrogels, as well as surface functionalization strategies using molecules such as hyaluronic acid (HyA). These approaches enhance mechanical performance, modulate degradation behavior, and promote cellular infiltration and vascularization, thereby supporting more effective tissue regeneration [36]. Mousavi Nejad et al. (2021) [13] fabricated PCL-based scaffolds in an in vitro study aimed at promoting dentin–pulp complex regeneration. Two scaffold types were developed: PCL incorporated with bioactive glass (BG) and PCL functionalized with HyA. Human dental pulp stem cells (hDPSCs) were used to evaluate cellular behavior and differentiation potential. The results demonstrated that BG improved mechanical properties, surface roughness, and scaffold bioactivity, while HyA increased hydrophilicity and enhanced cell adhesion. Moreover, the expression of odontogenic markers—dentin sialophosphoprotein (DSPP), osteocalcin (OCN), and dentin matrix protein 1 (DMP-1)—was significantly upregulated in these systems [13]. Huang et al. (2018) [37] developed a PCL-based scaffold loaded with bone morphogenetic protein-2 (BMP-2) incorporated into mesoporous calcium silicate (MesoCS). Using hDPSCs, the study demonstrated that PCL combined with calcium-based compounds exhibits significant odontogenic inductive potential. Enhanced cell adhesion, proliferation, and alkaline phosphatase (ALP) activity were observed in mesoporous scaffolds. Although primarily applicable to hard tissue regeneration, this system may also facilitate blood clot stabilization and promote odontogenesis in reparative endodontic therapies [37].

Polylactic acid (PLA) represents another class of thermoplastic polymers extensively investigated in tissue engineering. It is widely used in 3D printing for regenerative endodontics due to its excellent biocompatibility and biodegradability. Although PLA exhibits a controllable degradation rate, it may induce non-bacterial local inflammatory responses due to the release of acidic degradation byproducts [38,39]. Hsiao et al. (2020) demonstrated that PLA-based scaffolds, both alcohol-treated and coated with poly-L-lysine (PLL), improved hDPSC adhesion and did not induce significant local inflammatory responses, suggesting partial neutralization of acidic degradation products in vivo [39]. Chen et al. (2021) [40] applied hydroxyapatite (HA) coatings to PLA scaffolds in an in vivo canine model. The results revealed significantly enhanced mineralization in the experimental group, where scaffolds were seeded with dental pulp stem cells, compared to acellular controls. These findings highlight the capacity of dental pulp stem cells to undergo lineage-specific differentiation on HA/PLA substrates. However, a key limitation was the slow degradation rate, as scaffolds were not fully resorbed even after nine months [40].

### 3.2. Biomimetic Bioinks

In contrast to conventional 3D printing, which primarily employs synthetic materials for structural purposes with subsequent biological functionalization, bioprinting relies on specially designed materials known as bioinks. These materials enable the direct incorporation of living cells and bioactive factors during the layer-by-layer fabrication process. Bioinks may consist of natural polymers such as collagen, gelatin, alginate, hyaluronic acid (HyA), chitosan, and fibrin, as well as synthetic polymers such as polyethylene glycol dimethacrylate (PEGDMA) or copolymers based on polyethylene glycol (PEG) and polypropylene glycol (PPG). To optimize rheological and biological performance, chemically modified biopolymers are frequently employed, including gelatin methacryloyl (GelMA) and methacrylated hyaluronic acid (HAMA). In many formulations, these components are combined with bioactive bioceramics, contributing to the formation of stable three-dimensional constructs and enhancing cell–material interactions that mimic the native extracellular matrix [41,42]. Natural polymer-based bioinks represent the most widely used class in bioprinting due to their similarity to the native extracellular matrix and biological microenvironment. Among these, type I collagen is one of the most extensively used materials in regenerative endodontics, as it promotes dental pulp stem cell proliferation and upregulates genes associated with odontogenic differentiation, making it highly suitable for pulp regeneration applications [43,44,45]. Duarte Campos et al. (2020) [46] developed a bioink composed of type I collagen and agarose in an in vitro study. The results confirmed the biocompatibility of hydrogel-based constructs with dental pulp stem cells and demonstrated the formation of functional vascular networks, highlighting the potential of bioprinting approaches for in situ dental applications [46].

Despite their excellent biocompatibility, natural polymers often exhibit limited mechanical strength. Therefore, their combination with synthetic polymers provides improved mechanical stability and tunable physical properties [41]. Recent research has focused on hybrid hydrogels that integrate the biological advantages of natural polymers with the controllable characteristics of synthetic materials. Gelatin methacryloyl (GelMA) is one of the most extensively studied biomaterials in regenerative dentistry. At the molecular level, gelatin contains arginine–glycine–aspartic acid (RGD) sequences that promote cell adhesion, as well as sequences susceptible to matrix metalloproteinases (MMPs), enabling matrix remodeling and facilitating cell migration. Chemical modification of gelatin through the introduction of methacrylate groups allows the formation of stable crosslinked hydrogels suitable for bioprinting applications [47]. Monteiro et al. (2016) [48] developed a biomimetic 3D tooth bud model consisting of epithelial and mesenchymal cell layers combined with enamel organ- and pulp organ-like structures fabricated from GelMA hydrogels. In vitro analyses demonstrated the expression of key developmental markers, including Sonic Hedgehog (SHH), BMP2, and Runt-related transcription factor 2 (RUNX2), indicating active epithelial–mesenchymal interactions. Subsequent in vivo studies, involving subcutaneous implantation in a rat model, revealed the formation of mineralized tissues and sustained expression of differentiation markers, reflecting processes relevant to dentin formation and pulp regeneration [48]. In another in vivo study, Cunha et al. (2023) [49] applied GelMA hydrogels in direct contact with dental pulp tissue. The results demonstrated the formation of organized pulp-like tissue, tertiary dentin, tubular and atubular dentin, and neovascularization, suggesting that GelMA represents a promising material for regenerative pulp therapies and dentin formation [49].

Overall, hybrid biomaterial systems currently appear more promising for clinical translation than single-component scaffolds because they better reproduce the balance between mechanical stability and biological activity required for pulp regeneration [41]. Pure thermoplastic polymers such as PCL or PLA provide structural integrity but exhibit limited intrinsic bioactivity, whereas natural hydrogels support cell viability and differentiation but often lack sufficient mechanical stability [34,35]. Accordingly, composite bioinks integrating synthetic polymers, extracellular matrix-derived materials, and bioactive ceramics may represent the most clinically relevant strategy for achieving both structural support and functional tissue integration.

## 4. Stem Cell Types for 3D Biofabrication of Dental Tissues

A fundamental component of modern tissue regeneration strategies is the use of stem cells, characterized by their capacity for self-renewal and to generate progenitor cells that can differentiate into multiple specialized cell types. In the context of dental tissue reconstruction, the selection of an appropriate cell population, compatible with the biological characteristics of the target tissue, represents a critical determinant for achieving a predictable regenerative response, given the variability in differentiation potential among different stem cell types [50,51].

### 4.1. Origin and Types of Dental and Non-Dental Stem Cells

From the perspective of origin, the literature describes multiple populations of mesenchymal stem cells (MSCs) of dental origin, including dental pulp stem cells (DPSCs), stem cells from human exfoliated deciduous teeth (SHED), dental follicle progenitor cells (DFPCs), tooth germ progenitor cells (TGPCs), stem cells from the apical papilla (SCAP), periodontal ligament stem cells (PDLSCs), alveolar bone marrow stromal cells (ABMSCs), and gingiva-derived mesenchymal stem cells (GMSCs) [52,53]. In parallel, dental tissue engineering research has explored the use of non-dental stem cell sources, such as bone marrow-derived mesenchymal stem cells (BMSCs), human umbilical vein endothelial cells (HUVECs), amniotic fluid stem cells (AFSCs), and adipose-derived stem cells (ADSCs), due to their regenerative and angiogenic potential [27]. Stem cells of oral origin are considered particularly promising for pulp regeneration due to their enhanced capacity to differentiate into cell types specific to the dental microenvironment. Among the investigated stem cell populations, dental pulp stem cells (DPSCs) and stem cells from the apical papilla (SCAPs) currently appear the most promising for translational regenerative endodontics due to their odontogenic commitment, accessibility, and demonstrated angiogenic and neurogenic potential. Nevertheless, substantial variability persists regarding donor age, cell isolation protocols, expansion conditions, and differentiation capacity, which continues to limit reproducibility and large-scale clinical standardization [54,55]. Nevertheless, the survival, proliferation, and controlled differentiation of stem cells remain major challenges in dentin–pulp complex regeneration, given the structural complexity and cellular heterogeneity of this tissue. Although the formation of vascularized tissue is frequently reported in experimental studies, the predictable regeneration of a fully functional pulp—characterized by proper innervation and organized tubular dentinogenesis—remains difficult to achieve [56].

### 4.2. Cell Viability, Microenvironment, and Growth Factors

The success of regenerative processes is strongly influenced by the biological properties of the stem cell populations used, including their source-dependent differentiation potential, which underscores the importance of appropriate cell selection for functional tissue regeneration [57]. In this regard, dental mesenchymal stem cells exhibit multipotent differentiation capacity, being able to generate osteogenic/odontogenic and neurogenic lineages, thereby contributing to the regeneration of both structural and functional components of the dental pulp [58]. A major advantage of autologous stem cells is their high immunological compatibility and reduced risk of immune rejection, attributable to their inherent immunomodulatory properties [59]. To maintain stem cell viability and regenerative potential, the presence of a favorable pulp microenvironment is essential. This includes appropriate biochemical, physical, and mechanical conditions, such as controlled oxygen levels, interactions with the extracellular matrix, and biomechanical stimuli that support cell proliferation and differentiation [60,61]. In addition to the supportive microenvironment, growth factors play a crucial role in regulating stem cell behavior. They stimulate cell proliferation, migration, and differentiation, thereby enhancing the efficiency of regenerative processes [62]. Numerous growth factors have been identified as key regulators of dental pulp stem cell activity. For example, Yang et al. (2015) demonstrated that basic fibroblast growth factor (bFGF) promotes dentinogenesis, angiogenesis, and neurogenesis [63]. Furthermore, factors such as vascular endothelial growth factor (VEGF), bone morphogenetic proteins (BMP-2, BMP-4, BMP-7), transforming growth factor beta-1 (TGF-β1), fibroblast growth factor-2 (FGF-2), and platelet-derived growth factor (PDGF) are frequently incorporated into regenerative strategies to stimulate proliferation, induce odontogenic differentiation, and promote vascular network formation [62,64]. In this context, restoring pulp functionality requires not only structural reconstruction but also the re-establishment of a microenvironment capable of supporting metabolic exchange and biological signaling. Vascularization and innervation are essential components, contributing to tissue homeostasis, sensory function, and the delivery of nutrients and oxygen to newly formed cells [56]. Duarte Campos et al. (2020) [46] utilized DPSCs in combination with HUVECs and angiogenic growth factors such as VEGF to stimulate neovascularization. Bioprinting enabled precise spatial positioning of cells and growth factors, facilitating controlled vascular regeneration, which is essential for restoring pulp function [46]. Similarly, Qian et al. (2023) demonstrated that GelMA microspheres loaded with human DPSCs promoted both angiogenesis and neurogenesis during pulp tissue regeneration, as evidenced by the expression of neuronal markers such as microtubule-associated protein 2 (MAP2) and vascular markers such as cluster of differentiation 31 (CD_31_) [65].

## 5. In Vitro Evaluations

In vitro studies represent the initial stage in the evaluation of bioprinting strategies, providing a controlled framework for analyzing cell viability and proliferation, scaffold biocompatibility, and the expression of odontogenic differentiation markers, as well as for investigating the influence of the mechanical and biochemical microenvironment on cellular behavior. A summary of the main bioprinting systems, materials, and fabrication parameters used across the analyzed studies is presented in Table 1.

The analyzed data indicate that, under optimized printing parameters, the fabrication technology itself is not the primary determinant of cell survival. Where reported, post-printing viability frequently exceeds 90% across most systems, including fibrin-based bioinks (≈3 × 10^6^ cells/mL) and alginate–dentin constructs (0.8 × 10^6^ cells/mL). However, significant variations arise depending on composition. For example, viability values exceeding 95% have been reported in fibrin–gelatin bioinks incorporating demineralized dentin matrix, compared to approximately 65% in non-functionalized alginate, highlighting the critical role of matrix bioactivity [66,67,68].

In contrast, scaffold composition exerts a decisive influence on subsequent biological responses. Bioinert materials such as PCL or plain alginate support baseline cell viability but induce limited differentiation. The incorporation of bioactive components—including bioactive glass (BG), calcium silicate, dentin matrix, or Biodentine (BD)—results in consistent increases in DSPP and DMP-1 expression and enhanced mineralization. For instance, increases of up to 6–11-fold in calcium deposition have been reported in BD/PCL scaffolds compared to controls, alongside the overexpression of odontogenic markers in PCL/BG and demineralized dentin matrix powder (DDMp) systems [13,68,69]. Hybrid formulations such as gelatin methacryloyl/alginate/cellulose nanocrystals (GelMA/Alg/CNC) have demonstrated up to a 2.2-fold increase in odontogenic marker expression in optimized variants, confirming the regulatory role of the cell–matrix biochemical interface in differentiation [70]. The mechanical and compositional properties of the scaffold matrix act synergistically in directing cellular differentiation. Scaffolds with higher stiffness and the ability to release bioactive ions (Ca^2+^, Si^4+^) promote mineralization and odontogenic differentiation, whereas hydrogels with lower stiffness (≈1–2 kPa), closer to that of native pulp tissue, support a pulp-like phenotype and enhanced cellular proliferation. In this context, calcium silicate-enriched bioinks have demonstrated increased cell viability and odontogenic marker expression in an ion-dependent manner, suggesting that the release of bioactive ions is a key regulator of differentiation [67,71].

Scaffold architecture also contributes significantly to biological performance. Filament diameters in the range of 200–500 μm and intermediate pore sizes (≈400–700 μm) have been associated with improved cell adhesion and alkaline phosphatase (ALP) activity. For example, pore sizes of approximately 421 μm have been shown to induce higher ALP activity compared to larger structures (~700 μm), indicating the existence of an optimal range for cell–matrix interactions [13,72]. Additionally, surface functionalization through increased roughness and hydrophilicity (e.g., via plasma treatment and hyaluronic acid coating) significantly enhances cell adhesion and promotes a pulp-like phenotype [13]. In the context of pulp regeneration, angiogenic potential represents a critical factor. Co-culture systems of human dental pulp stem cells (hDPSCs) and human umbilical vein endothelial cells (HUVECs) have demonstrated the formation of CD31-positive vascular networks, with superior performance observed in fibrin-based hydrogels compared to collagen, where vascular tube length was significantly greater (*p* < 0.05), highlighting the importance of scaffold composition in supporting angiogenesis [46].

Overall, in vitro data indicate that, once technical parameters are optimized, biological outcomes are predominantly determined by the three-dimensional microenvironment, bioactive composition, mechanical properties, and scaffold architecture, all of which actively modulate the behavior and differentiation of dental stem cells. The corresponding in vitro biological outcomes, including cell viability, differentiation, and mineralization responses, are summarized in Table 2.

## 6. In Vivo Studies

In vivo studies are essential for evaluating the clinical applicability of bioprinting strategies, as they integrate complex processes such as vascularization, immune response, and tissue remodeling. Experimental models, including ectopic, orthotopic, and critical-sized bone defect models, enable the differential analysis of these processes and provide valuable insights into construct behavior under physiologically relevant conditions. An overview of the in vivo bioprinting constructs, materials, and fabrication approaches is presented in Table 3.

In ectopic subcutaneous animal models, construct performance is primarily determined by bioactive composition and porous architecture. GelMA hydrogels with microporosity (~20–50 μm) and elasticity of approximately 40 kPa have been shown to support cell viability and induce mechanotransduction activation (Yes-associated protein, YAP), which correlates with significant increases in osteogenic/odontogenic markers (RUNX2, OCN, DSPP), along with effective tissue integration and cellular infiltration [75]. Similarly, scaffolds based on decellularized extracellular matrix/beta-tricalcium phosphate (dECM/β-TCP) have demonstrated a critical balance between bioactivity and cytocompatibility. Optimal concentrations (~20 wt% β-TCP) maintained cell viability at approximately 96–97%, whereas higher concentrations reduced cytocompatibility, suggesting the existence of a threshold for stiffness and mineral content [76]. Orthotopic models further highlight the importance of controlled three-dimensional organization. The implantation of GelMA microspheres loaded with dental pulp stem cells (DPSCs) resulted in complete regeneration of vascularized pulp-like tissue along the entire root canal length within 4–8 weeks, accompanied by odontoblast-like organization and increased expression of angiogenic and neurogenic markers (VEGFα, CD31, microtubule-associated protein 2 (MAP2), growth-associated protein 43 (GAP43)). In contrast, cell delivery in suspension resulted in limited and non-uniform regeneration, indicating that a defined three-dimensional architecture is essential for maintaining cell viability and achieving functional integration [65]. In critical-sized bone defect models, 3D-printed hydroxyapatite/polylactic acid (HyA/PLA) scaffolds loaded with dental pulp stem cells demonstrated significant increases in mineralized volume and the formation of dentin- and bone-like structures in long-term evaluations (up to 9 months), as confirmed by micro-computed tomography (micro-CT) and histological analyses. However, partial scaffold degradation suggests an imbalance between resorption rate and tissue formation, highlighting the need for optimization of biomaterial degradation kinetics [40]. A consistent finding across the analyzed studies is the critical role of vascularization. Microporous scaffolds and dECM-based systems have demonstrated increased vascular density and significant VEGF expression without marked inflammatory responses, indicating a favorable microenvironment for tissue integration and functional regeneration [75,76]. A summary of the in vivo experimental models and functional outcomes, including tissue regeneration and vascularization, is presented in Table 4.

Overall, in vivo data confirm that three-dimensional architecture and scaffold bioactivity are the primary determinants of effective regeneration. Once technical parameters are optimized, the bioprinting technology itself becomes a secondary factor, while material composition, mechanical properties, and cellular organization ultimately dictate biological outcomes and clinical translational potential.

## 7. Challenges and Future Directions

Three-dimensional bioprinting for dentin–pulp complex regeneration has demonstrated remarkable progress in preclinical models, including high cell viability, enhanced odontogenic marker expression, and the formation of vascularized pulp-like tissue. However, clinical translation remains limited due to methodological variability, the lack of standardized performance criteria, and insufficient long-term functional evaluation, all of which affect the comparability and predictability of outcomes [65,67,75].

### 7.1. Bioink Standardization and the Three-Dimensional Microenvironment

A major obstacle to further progress is the variability of bioinks employed. Materials based on natural or synthetic polymers differ significantly in terms of mechanical properties, degradation kinetics, and bioactive factor release profiles, complicating the comparison of biological outcomes across experimental systems [13,46,77]. The development of an instructive microenvironment capable of maintaining a pulp-like phenotype while ensuring structural stability remains a key challenge. Materials with high stiffness promote mineralization but may hinder cellular infiltration and vascularization, whereas low-stiffness materials, although closer to native pulp tissue, often exhibit limited architectural stability [67,70,78]. Furthermore, scaffold degradation must be temporally synchronized with tissue regeneration. Rapid degradation may compromise cellular organization, while slow degradation may impair physiological remodeling. In this context, the controlled and sequential release of bioactive factors (e.g., BMP-2, Ca^2+^/Si^4+^ ions) requires a transition from empirical approaches toward predictive strategies based on well-characterized kinetics and standardized evaluation models [37,68].

### 7.2. Functionality-Vascularization and Reinnervation

The restoration of pulp functionality requires the reconstruction of both vascular and neural networks to support metabolic activity and sensory function. Although hDPSC–HUVEC co-culture systems have demonstrated vascular-like structures in vitro, the integrity and perfusability of these networks in vivo remain difficult to control and are often inconsistent across experimental models [46]. Advanced biofabrication strategies, including the 3D bioprinting of vascularized tissues, have demonstrated the generation of perfusable networks in thick constructs, suggesting promising approaches to overcome these limitations [79]. The coordinated regulation of angiogenesis, neurogenesis, and mineralization is essential to prevent fibrosis and premature mineralization [65,75]. Emerging strategies include the incorporation of perfusable microchannels, multimaterial bioprinting with precise spatial cell patterning, and the use of axon-guiding biomaterials to recreate the functional architecture of native pulp tissue [79].

### 7.3. Clinical Challenges and Preclinical Translation

Regeneration of the dentin–pulp complex faces challenges specific to the endodontic environment, including the narrow and complex geometry of root canals, the presence of microbial biofilms, and chronic inflammatory conditions. These factors directly influence material stability, stem cell behavior, and signaling pathways involved in odontogenic differentiation [80]. Furthermore, many experimental studies rely on ectopic implantation or small-animal models that only partially reproduce the anatomical confinement, microbial contamination, chronic inflammation, vascular restriction, and biomechanical conditions characteristic of human endodontic disease. As a result, the regenerative outcomes observed in these models may overestimate the translational potential of current bioprinting strategies [75,80]. Pulpal blood supply is restricted by vascular access through the apical foramen, which may result in hypoxic conditions, reduced cell viability, and incomplete tissue maturation [75]. Hydrogel-based materials, although highly conformable and suitable for minimally invasive delivery, often exhibit limited mechanical stability, whereas thermoplastic scaffolds provide structural support but may be difficult to position within complex canal anatomies [65]. These limitations highlight the need for hybrid strategies that combine mechanical integrity with biological functionality.

### 7.4. Control of Three-Dimensional Architecture and Cellular Distribution

Controlled three-dimensional organization is a key determinant of regenerative performance. Layer-by-layer bioprinting enables uniform cell distribution and more homogeneous differentiation compared to cell suspension delivery, which often results in heterogeneous or incomplete tissue formation [65]. Architectural parameters, including porosity, microscale resolution, and structural gradients, significantly influence cell adhesion and enzymatic activity (e.g., alkaline phosphatase). The integration of these features into clinically scalable systems requires advanced multimaterial printing techniques and personalized digital design approaches capable of replicating the heterogeneity of the native pulp microenvironment [13,43].

Notably, direct comparative studies specifically isolating the influence of printing modality from biomaterial composition remain scarce. Most currently available studies simultaneously modify multiple variables, including scaffold composition, crosslinking strategy, pore architecture, and cell type, making it difficult to determine the independent contribution of the fabrication method itself to regenerative outcomes.

### 7.5. Functional Validation and Long-Term Safety

Most studies primarily evaluate early biological indicators such as cell viability, gene expression, and mineralization, which, although informative, do not fully reflect clinical functionality. Importantly, most currently reported regenerative outcomes are based on surrogate biological markers, including ALP activity, DSPP/DMP-1 expression, and mineralization assays. While these indicators suggest odontogenic differentiation, they do not necessarily demonstrate the regeneration of a fully functional dentin–pulp complex capable of maintaining long-term vascular perfusion, sensory innervation, immune competence, and physiological dentin turnover under clinical conditions [13,68,69]. Consequently, future studies should prioritize functional validation under clinically relevant conditions, including sensory responsiveness, pulp homeostasis, and long-term tissue stability. Notably, clinical evidence supporting predictable long-term functional regeneration using bioprinted dentin–pulp constructs remains extremely limited, highlighting a major translational gap [81].

Beyond biological validation, clinical translation remains limited by substantial regulatory and manufacturing challenges. The combination of biomaterials, living cells, and bioactive molecules complicates regulatory approval and requires rigorous standardization of bioink formulation, crosslinking, sterilization, and printing parameters to ensure reproducibility across production batches. In addition, scaffold degradation kinetics must be synchronized with tissue remodeling, as both premature degradation and prolonged material persistence may impair regeneration. Collectively, these limitations underscore the need for standardized evaluation criteria and robust long-term preclinical validation before clinical implementation can be achieved.

### 7.6. Future Directions

Future perspectives in dentin–pulp complex bioprinting are centered on several key directions:

- Smart bioinks and biodynamic microenvironments: materials capable of dynamically adapting their mechanical properties and degradation behavior in response to local biological cues, thereby synchronizing scaffold remodeling with tissue regeneration.
- Prevascularization and neurovascular integration: the development of perfusable microchannels, endothelial cell patterning, and axon-guiding biomaterials to enable functional integration with host vascular and neural systems.
- In situ bioprinting and clinical personalization: direct fabrication within the root canal, supported by real-time imaging, robotic guidance, and AI-assisted design optimization, enabling patient-specific adaptation to anatomical and pathological conditions.
- Integrated clinical translation: the establishment of standardized protocols, reproducible outcome measures, scalable manufacturing processes, regulatory compliance, and cost-effectiveness, all of which are essential for integrating bioprinting into routine endodontic practice.
- Among currently investigated strategies, extrusion-based bioprinting combined with hybrid hydrogels, particularly GelMA-based systems incorporating dental pulp stem cells, represents one of the most promising approaches for translational application. This strategy benefits from manufacturing scalability, compatibility with clinically relevant biomaterials, and the ability to support vascularized pulp-like tissue formation in orthotopic large-animal models. However, further validation under standardized and clinically relevant conditions is required to confirm long-term functional performance and translational feasibility. By integrating these directions with fundamental principles of tissue regeneration, the field is progressing toward the restoration of fully functional dental pulp, moving beyond purely structural reconstruction.

## 8. Materials and Methods

This review was conducted based on a structured literature search to ensure transparency and reproducibility. Studies were identified through searches in the PubMed database and Google Scholar. Google Scholar was additionally used to retrieve peer-reviewed articles indexed in major scientific publishers, including Elsevier (ScienceDirect) and MDPI. The search strategy employed combinations of keywords such as “3D bioprinting”, “dentin–pulp complex”, “biomaterials”, “bioink”, “scaffolds”, “tissue engineering”, “regenerative dentistry”, “pulp regeneration,” and “stem cells”. The search was limited to articles published between 2015 and 2025. After removal of duplicates, studies were screened based on titles and abstracts for relevance. Full-text articles were then assessed for eligibility according to predefined inclusion and exclusion criteria. Inclusion criteria comprised original in vitro, in vivo, and preclinical studies focusing on 3D bioprinting strategies for dentin–pulp complex regeneration. Exclusion criteria included non-English publications, conference abstracts, reviews without original data, and studies lacking relevant biological outcomes. Although the study selection process followed PRISMA-inspired principles, this review remains narrative in design. Therefore, a formal systematic quality assessment and a PRISMA flow diagram were not performed.

During the preparation of this manuscript/study, the authors used GenAI tools (e.g., ChatGPT [OpenAI, GPT-5.5]) for the purposes of language editing and English translation, as well as to support the creation of Figure 1, Figure 2, Figure 3 and Figure 4. The authors have reviewed and edited the output and take full responsibility for the content of this publication.

## 9. Conclusions

Three-dimensional bioprinting represents a promising approach for dentin–pulp complex regeneration, offering precise control over the architecture and composition of biomimetic constructs. Current evidence suggests that scaffold composition and the engineered three-dimensional microenvironment may exert a stronger influence on biological outcomes than the printing modality itself. However, direct comparative studies isolating these variables remain limited, and definitive conclusions cannot yet be established due to methodological heterogeneity across studies.

The main challenges are increasingly related to the optimization of the microenvironment, the standardization of bioink formulations, and the validation of long-term functional outcomes, rather than the feasibility of bioprinting approaches. In this context, bioprinting should be regarded not merely as a fabrication technique but as a platform for the controlled modulation of biological processes involved in dental pulp regeneration, with promising long-term potential for clinical translation, although current evidence remains predominantly experimental and insufficient to support predictable clinical implementation.

## Figures and Tables

**Table 1:** In vitro studies on 3D bioprinted scaffolds for dental tissue engineering.

Author/Year	Tissue Type	Material/Bioink (Experimental)	Material/Bioink (Control)	Cell Type	Printing Method	Bioprinting Conditions	Evidence Level/Model Type
**Mousavi Nejad et al. (2021)** [13]	Dentin–pulp	PCL + 45S5 BG/PCL + HyA	PCL	hDPSC	Extrusion-based bioprinting	Bioprinter N_2_ (3DPL Co., Tehran, Iran): 90 °C; 600 kPa; 2 mm/s; PCL:BG 70:30; plasma (100 W, 0.6 mbar, 40 kHz, 10 min); HyA coating; freeze-drying (−58 °C, 0.5 Torr, 24 h).	In vitro/ex vivo
**Han et al. (2019)** [66]	Dentin–pulp	Fibrin (F5-F20) + PCL	Control (fibrin formulations, F5–F20)	hDPSC	Extrusion-based bioprinting	Custom 3D bioprinter (3-axis, multi-cartridge): nozzle 100–200 µm; speed 0.83–2.33 mm/s; dispensing 34.55–138.21 nL/s; fibrinogen 5–50 mg/mL.	In vitro/ex vivo
**Athirasala et al. (2018)** [67]	Dentin–pulp	Alginate–dentin (3% alginate; 2:1/1:1/1:2 Alg:Dent ratios)	Control (Alg:Dent ratios)	SCAPs	Extrusion-based bioprinting	Hyrel 3D (Norcross, GA, USA): coaxial nozzle 26G/19G; flow 45 µL/min; feed 0.5–0.8; grids 20 × 20 mm; 4-layer constructs (15 × 15 mm).	In vitro/ex vivo
**Duarte Campos et al. (2020)** [46]	Pulp tissue	Collagen type I-agarose (0.2%/0.5%)	Fibrin (0.5%) or Collagen type I (0.3%) (cell-free)	hDPSC + HUVEC	Inkjet (drop-on-demand)	DropGun (BlackDrop, Darmstadt, Germany): nozzle 300 µm; pressure 25–250 kPa; droplet 20–600 nL; frequency ≤ 1000 Hz; layer ~100 µm; alginate 0.5%; collagen 0.2%; RT printing.	In vitro/ex vivo
**Jiang et al. (2021)** [43]	Dentin	Collagen type I/silk fibroin (CSF1–3)	Control (CSF formulations)	hDPSC	Extrusion-based bioprinting	OrganP 1800 (Chongqing, China): nozzle 260 µm; speed 2–10 mm/s; layer 320 µm; height 0.7 mm; platform 20 °C; collagen/silk fibroin (1:1).	In vitro/ex vivo
**Huang et al. (2018)** [37]	Dentin	MesoCS + BMP-2	Control (non-mesoporous CS scaffold)	hDPSC	Extrusion-based bioprinting	Bio-Scaffolder 3.1 (GeSiM, Radeberg, Germany): nozzle 400–500 µm; pressure 400–500 kPa; speed 1–5 mm/s; layer height 300 µm; spacing 500–600 µm; 7–16 layers; 90° orientation.	In vitro/ex vivo
**Han et al. (2021)** [68]	Dentin	Fibrinogen + DDMp	Control (no DDMp)	hDPSC	Extrusion-based bioprinting	Custom multi-cartridge bioprinter: nozzle 300 µm; speed 0.08–5.33 mm/s; extrusion 34.3 µL/min; layer width 400 µm; layer height 150 µm.	In vitro/ex vivo
**Ho et al. (2018)** [69]	Dentin	Biodentine + PCL	PCL	hDPSC	Extrusion-based bioprinting	BioScaffolder 3.1 (variant): nozzle 500 µm; pressure 500 kPa; speed 1–5 mm/s; layer height 300 µm; spacing 500 µm; 7 layers; 90° orientation.	In vitro/ex vivo
**Li et al. (2024)** [70]	Dentin	GelMA/Alg/CNC formulations (0–5% Alg; inverse CNC gradient, 6 groups)	Control (GelMA/Alg/CNC ratio formulations)	hDPSC	Extrusion-based bioprinting	3D Discovery Evolution (regenHU, Villaz-Saint-Pierre, Switzerland): nozzle 260 µm; temperature 20 °C; UV 365 nm (5 min); Ca^2+^ crosslinking 0.1 M (1 h).	In vitro/ex vivo
**Lin et al. (2021)** [71]	Dentin	GelMA + CS (5–10%)	GelMA	hDPSC	Extrusion-based bioprinting	BioX (CELLINK, Göteborg, Sweden): nozzle 30G; pressure 180 kPa; speed 20 mm/s; UV curing 405 nm (90 s).	In vitro/ex vivo
**Choi et al. (2022)** [72]	Dentin	GelMA + MTA	GelMA	hDPSC	Extrusion-based bioprinting	Rokit INVIVO 3D: nozzle 200 µm; speed 10 mm/s; pressure 90 kPa; bed 4 °C; UV 365 nm (5–10 min); ethanol 70% (30 min); lyophilization (−70 °C, 24 h).	In vitro/ex vivo
**Yeh et al. (2022)** [73]	Dentin	MSCS/2% Quercetin/PCL	MSCS + PCL	hDPSC	Extrusion-based bioprinting	Bio-Scaffolder 3.1 (GeSiM, Radeberg, Germany): nozzle 400 µm; pressure 400 kPa; layer height 300 µm; spacing 600 µm.	In vitro/ex vivo
**Yu et al. (2019)** [74]	Dentin	Alg/Gel + hDPSCs	hDPSCs (no extract)	hDPSC	Extrusion-based bioprinting	Bioplotter (ETEC, Wendlingen am Neckar, Germany): nozzle 400 µm; N_2_ pressure 20 kPa; speed 2 mm/s; temperature 37 °C; platform 5 °C; 7 layers.	In vitro/ex vivo

PCL—polycaprolactone; BG—bioactive glass; 45S5 BG—45S5 bioactive glass composition; HyA—hyaluronic acid; hDPSC—human dental pulp stem cells; SCAPs—stem cells from the apical papilla; HUVEC—human umbilical vein endothelial cells; GelMA—gelatin methacrylate; MTA—mineral trioxide aggregate; CS—chitosan; CSF—collagen–silk fibroin; MesoCS—mesoporous calcium silicate; BMP-2—bone morphogenetic protein-2; DDMp—decellularized dentin matrix particles; MSCS—mesenchymal stem cells; Alg—alginate; Dent—dentin; F5–F20—fibrinogen formulations (5–20 mg/mL); N_2_—nitrogen gas; UV—ultraviolet; kPa—kilopascal; µm—micrometer; µL/min—microliter per minute; nL/s—nanoliter per second; RT—room temperature.

**Table 2:** In vitro evaluation of 3D bioprinted scaffolds for dental tissue engineering.

Author/Year	Tissue Type	Material/Bioink	Cell Type (Cell Density)	Assessment	Outcomes
**Mousavi Nejad et al. (2021)** [13]	Dentin–pulp	PCL + 45S5 BG/PCL + HyA	hDPSC (5 × 10 ^4^ cells/well)	Viability (MTT); morphology (FESEM/EDS); gene expression (RT-qPCR); surface roughness (AFM); mechanical testing (compression)	Viability > 90% (*p* < 0.01). PCL/BG enhanced odontogenic differentiation via RT-qPCR upregulation of ALP, DSPP, DMP-1, OCN and hydroxyapatite formation with improved mechanical strength. PCL/HyA improved adhesion, wettability (63°), and pulp-like phenotype (VEGF, HLA, CEMP1).
**Han et al. (2019)** [66]	Dentin–pulp	Fibrin + PCL	hDPSC (3 × 10^6^ cells/mL)	Viability (Live/Dead, alamarBlue); mineralization (ARS); differentiation (ALP); gene expression (RT-qPCR); morphology (SEM)	Viability > 90% (Day 4). Fibrin ≥ 10 mg/mL enhanced mineralization and odontogenic gene expression (DSPP, DMP1). F20 showed highest odontogenic potential; F5 favored pulp-like phenotype. Increased stiffness (1.5×) and reduced degradation (~50%).
**Athirasala et al. (2018)** [67]	Dentin–pulp	Alg:Dent	SCAPs (0.8 × 10^6^ cells/mL)	Rheology; mechanical testing; viability (Live/Dead); differentiation (ALP); gene expression (RT-qPCR)	Viability > 90% (1:1/1:2). Strong RT-qPCR upregulation of ALP and RUNX2 (up to 26×). Scaffold exhibited pulp-like stiffness (1–2 kPa) and supported odontogenic differentiation without external induction.
**Duarte Campos et al. (2020)** [46]	Pulp tissue	Collagen type I -agarose	hDPSC (3 × 10^6^ cells/mL) + HUVEC (3 × 10^6^ cells/mL) (co-culture)	Morphology/vasculogenesis (CD31 confocal); SEM; rheology; mechanical testing; printability analysis	Co-culture formed CD31+ vascular networks. Hydrogels supported vasculogenesis and structural stability up to 14 days. Fibrin-based systems enhanced vascular tube formation vs. collagen-based constructs.
**Jiang et al. (2021)** [43]	Dentin	Collagen type I/silk fibroin	hDPSC (1 × 10^5^ cells/scaffold)	Morphology (SEM); viability (CCK-8); differentiation (ALP); histology (HE)	CSF scaffolds enhanced adhesion and proliferation (*p* < 0.05), with highest ALP activity in CSF1 (*p* < 0.01). Multilayer growth observed; odontogenic differentiation confirmed, indicating dentin–pulp regenerative potential.
**Huang et al. (2018)** [37]	Dentin	MesoCS + BMP-2	hDPSC NR	Viability (PrestoBlue); morphology (SEM/TEM); mineralization (ARS); ALP; gene/protein expression (RT-qPCR/ELISA/WB); ion release (ICP-AES); mechanics	MesoCS increased proliferation (+37%, *p* < 0.05), ALP activity and odontogenic markers (OC, DMP-1, DSPP). Enhanced mineralization and BMP-2 release (~2×) with SMAD/ERK pathway activation.
**Han et al. (2021)** [68]	Dentin	Fibrinogen + DDMp	hDPSC (3 × 10^6^ cells/mL)	Viability (Live/Dead, AlamarBlue); mineralization (ARS); differentiation (ALP); gene expression (RT-qPCR); morphology (SEM)	Viability > 95%. DDMp reduced proliferation but significantly enhanced mineralization and odontogenic markers DSPP and DMP-1 (*p* < 0.01).
**Ho et al. (2018)** [69]	Dentin	Biodentine + PCL	hDPSC (1 × 10^4^ cells/mL)	Morphology (SEM); viability (PrestoBlue); ALP; protein expression (ELISA); mineralization (ARS)	BD/PCL enhanced proliferation (1.7×–1.3×, *p* < 0.05), ALP activity and OC secretion. Strong mineralization observed (up to 11.7× vs. control), confirming osteoinductive effect.
**Li et al. (2024)** [70]	Dentin	GelMA/Alg/CNC	hDPSC (1 × 10^6^ cells/mL)	Viability (Live/Dead, MTT); morphology (SEM, AFM); mechanics (compression/rheology); structure (XRD/TGA); differentiation (ALP); gene expression (RT-qPCR)	GelMA/Alg/CNC (GelMA-2A3C) showed highest viability and proliferation (*p* < 0.001), strongest ALP activity and upregulation of ALP, OPN and DSPP (1.5–2.2×), with superior mineralization among all formulations.
**Lin et al. (2021)** [71]	Dentin	GelMA + CS	hDPSC (5 × 10^6^ cells/mL)	Morphology (confocal/FTIR/XRD); rheology; ion release (ICP-AES); protein expression (ELISA/WB); ALP	CS/GelMA increased viability and proliferation (*p* < 0.05). CS10 showed highest mineralization and upregulation of ALP, DMP-1 and OCN (*p* < 0.01), driven by Si ion release.
**Choi et al. (2022)** [72]	Dentin	GelMA + MTA	hDPSC (5 × 10^4^ cells/well)	Morphology (SEM); viability (WST-1); gene expression (RT-qPCR); ALP; mineralization (ARS)	Cell viability comparable to control (*p* > 0.05). MTA-GelMA increased ALP activity and calcium deposition (*p* < 0.05) with upregulation of DSPP and DMP-1, indicating odontogenic differentiation.
**Yeh et al. (2022)** [73]	Dentin	MSCS/Quercetin/PCL	hDPSC (1 ×10^4^ cells/mL)	Morphology (TEM/SEM); mechanics (XRD); viability (PrestoBlue); protein expression (ELISA); mineralization (ARS)	MSCSQ increased viability (~15%, *p* < 0.05), proliferation and mineralization with upregulation of DSPP and DMP-1, supporting pulp–dentin regeneration.
**Yu et al. (2019)** [74]	Dentin	Alg/Gel + hDPSCs	hDPSC (1 × 10^6^ cells/mL/~5 × 10^4^ cells/scaffold)	Viability (Live/Dead, MTT, CCK-8); morphology (SEM); differentiation (ALP); gene expression (RT-qPCR); protein expression (WB)	Alg-Gel scaffolds enhanced proliferation (1.2–1.4×, *p* < 0.001), ALP activity and expression of ALP, OCN and DSPP, with increased mineralization and osteo/odontogenic differentiation.

PCL—polycaprolactone; BG—bioactive glass; 45S5 BG—45S5 bioactive glass composition; HyA—hyaluronic acid; hDPSC—human dental pulp stem cells; SCAPs—stem cells from the apical papilla; HUVEC—human umbilical vein endothelial cells; GelMA—gelatin methacrylate; MTA—mineral trioxide aggregate; CS—chitosan; CSF—collagen–silk fibroin; MesoCS—mesoporous calcium silicate; BMP-2—bone morphogenetic protein-2; DDMp—decellularized dentin matrix particles; MSCS—mesenchymal stem cells; Alg—alginate; CNC—cellulose nanocrystals; BD—Biodentine; MTT—3-(4,5-dimethylthiazol-2-yl)-2,5-diphenyltetrazolium bromide assay; WST-1—water-soluble tetrazolium assay; CCK-8—Cell Counting Kit-8; Live/Dead—live/dead viability assay; alamarBlue—resazurin-based viability assay; ARS—Alizarin Red S staining; ALP—alkaline phosphatase; RT-qPCR—reverse transcription quantitative polymerase chain reaction; ELISA—enzyme-linked immunosorbent assay; WB—Western blot; SEM—scanning electron microscopy; FESEM—field emission scanning electron microscopy; TEM—transmission electron microscopy; EDS—energy-dispersive spectroscopy; AFM—atomic force microscopy; XRD—X-ray diffraction; FTIR—Fourier-transform infrared spectroscopy; TGA—thermogravimetric analysis; ICP-AES—inductively coupled plasma atomic emission spectroscopy; HE—hematoxylin and eosin staining; CD31—cluster of differentiation 31 (endothelial marker); VEGF—vascular endothelial growth factor; HLA—human leukocyte antigen; CEMP1—cementum protein 1; DSPP—dentin sialophosphoprotein; DMP-1—dentin matrix protein 1; OCN/OC—osteocalcin; OPN—osteopontin; RUNX2—runt-related transcription factor 2; NR—not reported; kPa—kilopascal.

**Table 3:** Preclinical in vivo application of bioprinted constructs for dental tissue engineering.

Author/Year	Tissue Type	Material/Bioink (Experimental)	Material/Bioink (Control)	Cell Type	Printing Method	Printing Parameters	Evidence Level/Model Type
**Zhou et al. (2024)** [75]	Pulp Tissue	GelMA/dextran emulsion	Bulk GelMA hydrogel	hDPSCs/ HUVECs	Digital Light Processing (DLP) bioprinting	405 nm UV light; DMD-based patterning; 10 s/layer exposure; layer-by-layer photopolymerization; post-curing at 37 °C in PBS	In vivo ectopic
**Qian et al. (2023)** [65]	Pulp and Spinal Cord Tissue	GelMA hydrogel microspheres	2D-GelMA 2D-PS	hDPSCs	Digital Light Processing (DLP) bioprinting	405 nm UV light; 5% GelMA + 0.25% LAP; DLP-based layer-by-layer photopolymerization	In vivo ectopic + Orthotopic animal
**Chen et al. (2021)** [40]	Dentin-/Bone-like Tissue	HyA/PLA scaffold	HyA/PLA scaffolds (Cell-free)	DPSCs (canine-derived cell line)	Material extrusion–based 3D printing (FDM)	Ultimaker 2.0 Plus; 250 μm nozzle; 30 mm/s printing speed; 100 μm layer height; 200 °C nozzle; 60 °C bed; Cura 2.7 slicing	In vivo ectopic
**Kim et al. (2022)** [76]	Dentin Tissue	dECM/β-TCP composite	Collagen type 1/ β-TCP	hDPSCs	Pneumatic extrusion-based bioprinting	25 G nozzle (250 μm); 10 mm/s deposition speed; pneumatic extrusion; genipin crosslinking (1 mM, 30 min, 37 °C, 5% CO_2_)	In vivo ectopic

GelMA—gelatin methacrylate; HyA—hyaluronic acid; PLA—polylactic acid; dECM—decellularized extracellular matrix; β-TCP—beta-tricalcium phosphate; LAP—lithium phenyl-2,4,6-trimethylbenzoylphosphinate (photoinitiator); hDPSCs—human dental pulp stem cells; HUVECs—human umbilical vein endothelial cells; DPSCs—dental pulp stem cells; DLP—digital light processing; DMD—digital micromirror device; FDM—fused deposition modeling; PBS—phosphate-buffered saline; PS—polystyrene; UV—ultraviolet; °C—degrees Celsius; mm/s—millimeters per second; μm—micrometer; G—needle gauge; mM—millimolar; CO_2_—carbon dioxide.

**Table 4:** Preclinical in vivo evaluation of 3D bioprinted scaffolds for dental tissue engineering.

Author/Year	Tissue Type	Animal Model	Defect Area	Assessment	Outcomes
**Zhou et al. (2024)** [75]	Pulp Tissue	Immunodeficient mice	Subcutaneous implantation (ectopic; not explicitly reported)	CLSM, SEM, qPCR, ALP, IHC, CCK-8, transwell, H&E, tube formation, neurite assay, mechanical testing	DPGC hydrogels (~40 kPa, ~49 μm porosity) supported hDPSC viability and stemness (OCT4, NANOG, SOX2; *p* < 0.01) with YAP activation, while enhancing osteo/odontogenic differentiation (RUNX2, OCN, DSPP; *p* < 0.001), angiogenesis (VEGF, tube formation assays) and neurogenic responses (NGF, neurite outgrowth) after 4 weeks.
**Qian et al. (2023)** [65]	Pulp and Spinal Cord Tissue	Minipig, rat, nude mouse	Rat SCI: T10 complete spinal cord transection injury (~3 mm gap); mouse pulp model (apical foramen enlargement ~1 mm); minipig root canal model; subcutaneous degradation assay	CLSM, SEM, RT-qPCR, ALP, IF, CCK-8, transwell, H&E, micro-CT, motor scoring	Enabled functional recovery in SCI (BBB 8–9), reduced lesion size and upregulated GAP43/MAP2/GFAP. In pulp models, promoted vascularized pulp regeneration with Ki67+/OCT4+/SOX2+ cells, DSPP/DMP1 expression, angiogenesis (CD31/VEGFα) and neurogenesis (MAP2/GAP43), resembling native pulp at 4–8 weeks.
**Chen et al. (2021)** [40]	Dentin-/Bone-like Tissue	Beagle dogs	Bilateral mandibular defects (incisors 3 × 8 mm; premolars 6 × 8 mm)	H&E, Masson’s trichrome, IHC (DSPP, DMP1), micro-CT, histomorphometry, qRT-PCR	HyA/PLA scaffolds supported progressive mineralization and dentin/bone-like tissue formation over 6–36 weeks, with increased bone/dentin-like tissue volume and mineralized matrix density (*p* < 0.05). dDPSC-seeded scaffolds enhanced mineral deposition and remodeling, with partial degradation observed up to 9 months.
**Kim et al. (2022)** [76]	Dentin Tissue	Nude mice	Subcutaneous ectopic implantation (dorsal; incision depth not explicitly reported)	H&E, IF (DSPP, DMP1), qRT-PCR, ALP, ARS, MTT, cytoskeletal staining	dECM-based scaffolds enhanced angiogenesis (*p* < 0.05), odontogenic gene expression (DSPP, DMP1; *p* < 0.01) and mineralized matrix formation, promoting osteo/odontogenic differentiation and dentin-like tissue formation after 8 weeks.

hDPSC—human dental pulp stem cell; dDPSC—differentiated dental pulp stem cells; SCI—spinal cord injury; T10—thoracic vertebra 10; BBB—Basso, Beattie, Bresnahan locomotor rating scale; CLSM—confocal laser scanning microscopy; SEM—scanning electron microscopy; qPCR/RT-qPCR—quantitative/reverse transcription quantitative polymerase chain reaction; ALP—alkaline phosphatase; IHC—immunohistochemistry; IF—immunofluorescence; H&E—hematoxylin and eosin staining; ARS—Alizarin Red S staining; MTT—3-(4,5-dimethylthiazol-2-yl)-2,5-diphenyltetrazolium bromide assay; micro-CT—micro-computed tomography; DSPP—dentin sialophosphoprotein; DMP1—dentin matrix protein 1; OCN—osteocalcin; RUNX2—runt-related transcription factor 2; VEGF/VEGFα—vascular endothelial growth factor; CD31—cluster of differentiation 31; NGF—nerve growth factor; GAP43—growth-associated protein 43; MAP2—microtubule-associated protein 2; GFAP—glial fibrillary acidic protein; OCT4, NANOG, SOX2—stemness-associated transcription factors; YAP—yes-associated protein (Hippo pathway effector); Ki67—proliferation marker; HyA—hyaluronic acid; PLA—polylactic acid; dECM—decellularized extracellular matrix; kPa—kilopascal; μm—micrometer.

## Data Availability

No new data were created or analyzed in this study. Data sharing is not applicable to this article.

## Abbreviations

The following abbreviations are used in this manuscript:

AFM	Atomic Force Microscopy
ALP	Alkaline Phosphatase
Alg	Alginate
ARS	Alizarin Red S Staining
BBB	Basso, Beattie, Bresnahan Locomotor Rating Scale
BD	Biodentine
BG	Bioactive Glass
BMP-2	Bone Morphogenetic Protein-2
BMP-4	Bone Morphogenetic Protein-4
BMP-7	Bone Morphogenetic Protein-7
bFGF	Basic Fibroblast Growth Factor
Ca^2+^	Calcium Ion
CAD	Computer-Aided Design
CCK-8	Cell Counting Kit-8
CD31	Cluster of Differentiation 31
CEMP1	Cementum Protein 1
CLSM	Confocal Laser Scanning Microscopy
CNC	Cellulose Nanocrystals
CO_2_	Carbon Dioxide
CS	Chitosan
CSF	Collagen–Silk Fibroin
CT	Computed Tomography
dDPSC	Differentiated Dental Pulp Stem Cells
dECM	Decellularized Extracellular Matrix
DDMp	Decellularized Dentin Matrix Particles
DLP	Digital Light Processing
DMD	Digital Micromirror Device
DMP1	Dentin Matrix Protein 1
DSPP	Dentin Sialophosphoprotein
DPSC	Dental Pulp Stem Cell
EDX/EDS	Energy-Dispersive X-Ray Spectroscopy
EBP	Extrusion-Based Bioprinting
ELISA	Enzyme-Linked Immunosorbent Assay
FDM	Fused Deposition Modeling
FESEM	Field Emission Scanning Electron Microscopy
FGF-2	Fibroblast Growth Factor-2
FTIR	Fourier-Transform Infrared Spectroscopy
G (Gauge)	Needle Diameter Unit
GAP43	Growth-Associated Protein 43
GelMA	Gelatin Methacryloyl
GFAP	Glial Fibrillary Acidic Protein
H&E	Hematoxylin and Eosin Staining
HA	Hydroxyapatite
HAMA	Methacrylated Hyaluronic Acid
HE	Hematoxylin and Eosin Staining
HLA	Human Leukocyte Antigen
hDPSC	Human Dental Pulp Stem Cells
HUVEC	Human Umbilical Vein Endothelial Cells
HyA	Hyaluronic Acid
ICP-AES	Inductively Coupled Plasma Atomic Emission Spectroscopy
IF	Immunofluorescence
IHC	Immunohistochemistry
IJP	Inkjet-Based Bioprinting
kPa	Kilopascal
Ki67	Proliferation Marker
LAP	Lithium Phenyl-2,4,6-Trimethylbenzoylphosphinate
LIFT	Laser-Induced Forward Transfer
Live/Dead	Live/Dead Viability Assay
MAP2	Microtubule-Associated Protein 2
MesoCS	Mesoporous Calcium Silicate
MMPs	Matrix Metalloproteinases
MRI	Magnetic Resonance Imaging
MTA	Mineral Trioxide Aggregate
MTT	3-(4,5-Dimethylthiazol-2-Yl)-2,5-Diphenyltetrazolium Bromide Assay
μm	Micrometer
µL/min	Microliter Per Minute
MSCs	Mesenchymal Stem Cells
nL/s	Nanoliter Per Second
N_2_	Nitrogen Gas
NANOG	Homeobox Transcription Factor NANOG
NGF	Nerve Growth Factor
OC/OCN	Osteocalcin
OPN	Osteopontin
OCT4	Octamer-Binding Transcription Factor 4
PBS	Phosphate-Buffered Saline
PCL	Polycaprolactone
PDGF	Platelet-Derived Growth Factor
PEG	Polyethylene Glycol
PEGDMA	Polyethylene Glycol Dimethacrylate
PLA	Polylactic Acid
PLL	Poly-L-Lysine
PPG	Polypropylene Glycol
PS	Polystyrene
RT-qPCR	Reverse Transcription Quantitative Polymerase Chain Reaction
RGD	Arginine–Glycine–Aspartic Acid
RT	Room Temperature
RUNX2	Runt-Related Transcription Factor 2
SCAPs	Stem Cells from the Apical Papilla
SLA	Stereolithography
SHH	Sonic Hedgehog
Si^4+^	Silicon Ion
SEM	Scanning Electron Microscopy
SOX2	SRY-Box Transcription Factor 2
T10	Thoracic Vertebra 10
TGF-β1	Transforming Growth Factor Beta-1
TEM	Transmission Electron Microscopy
TGA	Thermogravimetric Analysis
TCP	Tricalcium Phosphate
β-TCP	Beta-Tricalcium Phosphate
UV	Ultraviolet
VEGF	Vascular Endothelial Growth Factor
WB	Western Blot
WST-1	Water-Soluble Tetrazolium Assay
XRD	X-Ray Diffraction
YAP	Yes-Associated Protein
